# (W/O/W) Double Emulsions-Filled Chitosan Hydrogel Beads for Topical Application

**DOI:** 10.3390/gels11070504

**Published:** 2025-06-27

**Authors:** Rui Sun, Yufeng Sun, Xiaoyan Tang, Juling Ji

**Affiliations:** Department of Pathology, Medical School of Nantong University, Nantong 226001, China; syf0817@ntu.edu.cn (Y.S.); 2213330010@stmail.ntu.edu.cn (X.T.)

**Keywords:** double emulsions, chitosan hydrogel beads, vitamin C, topical application, skin permeation

## Abstract

The aim of this study was to develop double emulsions-filled chitosan hydrogel beads for topical application and to elucidate their skin penetration behavior. Double emulsions were prepared by a two-step emulsification method, and double emulsions-filled chitosan hydrogel beads were prepared by the extrusion method. The structure, stability, and skin penetration behavior were investigated. The results of yield efficiency (above 80%) and microstructure observation confirmed the feasibility of the preparation method. After loading the hydrophilic active ingredients (vitamin C) into this system, the retention ratio after storage for 6 weeks increased by 77.6%. Furthermore, hydrogel beads could promote the permeation of hydrophilic active ingredients loaded in double emulsions. When the concentration of chitosan was 3% (*w*/*v*), the permeation coefficient of vitamin C from hydrogel beads exhibited an increase (1.7-fold) compared with double emulsions. This system could affect the orderliness of lipid structures in the stratum corneum. In addition, the results indicated that this system could be used for the topical delivery of hydrophobic active ingredients (quercetin) as well. This is the first report of chitosan bead stabilization of W/O/W emulsions, yielding a 2.6-fold increase in skin uptake of hydrophilic actives.

## 1. Introduction

Delivery systems are engineered to improve the efficacy of active ingredients through the use of suitable carriers [1]. The wall materials of delivery systems could act as a barrier to isolate the active ingredients from the external environment and optimize transport mechanisms [2]. Topical delivery systems offer the distinct benefit of releasing higher concentrations of active ingredients directly to the skin, avoiding dilution in circulation [3]. Owing to their remarkable advantages, including high biocompatibility, low toxicity, diverse structural designs, and extensive material sources, lipid-based delivery systems exhibit great potential for topical applications [4]. Consequently, they have garnered significant attention from both the pharmaceutical and cosmetic industries as a promising solution to address challenges associated with the topical application of active ingredients. Currently, lipid-based delivery systems have been widely applied in the delivery of hydrophilic and hydrophobic active ingredients [5].

(W/O/W) double emulsions are one of the lipid-based delivery systems that are suitable for encapsulating hydrophilic active ingredients, and they also enable the simultaneous loading of both hydrophilic and hydrophobic active ingredients [6]. The structure of (W/O/W) double emulsions can be described as aqueous droplets encapsulated within oil droplets, which are subsequently dispersed in the continuous aqueous phase [6]. The special multiple structure endows (W/O/W) double emulsions with unique properties. (W/O/W) double emulsions have the effect of improving the stability of hydrophilic active ingredients loaded in the inner aqueous phase [7]. Compared with the traditional (W/O) emulsions, (W/O/W) double emulsions have a stronger permeation-promoting ability for hydrophilic active ingredients, which is because their outer aqueous phase can promote skin hydration and thereby facilitate the transdermal permeation of hydrophilic active ingredients. Therefore, (W/O/W) double emulsions have been used for the topical delivery of hydrophilic active ingredients [8,9]. The study about (W/O/W) double emulsions loaded with fluorouracil indicated that (W/O/W) double emulsions increased the transdermal permeation amount of fluorouracil compared with (W/O) emulsions, and the infrared spectrum of the skin revealed that the permeation-promoting effect of (W/O/W) double emulsions may result from their ability to disrupt the structural integrity of the stratum corneum [10]. (W/O/W) double emulsions can also be used for transdermal delivery of biological macromolecules, such as polypeptides, proteins, and oligonucleotides [11,12,13]. The multiple structures of (W/O/W) double emulsions can effectively isolate the inner and outer aqueous phases, thereby providing a protective environment for biological macromolecules loaded in the inner aqueous phase.

The unique structure of double emulsions enables them to encapsulate, protect, and release hydrophilic active ingredients. However, this complex multiple structure also leads to the destabilization of double emulsions [7]. Compared with traditional emulsions, double emulsions exhibit poorer stability and a more complex instability mechanism due to the presence of two distinct interfaces and higher free energy [7]. Therefore, there is currently no industrialized preparation method available for stable double emulsions, which significantly limits their applications.

Hydrogel beads are granular hydrogels that exhibit superior dispersibility compared to large-volume bulk hydrogels [14]. They can serve as a semi-solid raw material for incorporation into products, enabling the encapsulation of active ingredients while maintaining the final physical state of the products [15]. By selecting an optimal addition level of hydrogel beads, the products can maintain excellent fluidity. Moreover, hydrogel beads can impart a unique appearance to the products, thereby enhancing consumer acceptance. This indicates a very broad application potential in industries such as food and cosmetics [16]. In recent years, double emulsions-filled hydrogel beads have been widely used to modulate the stability and optimize the performance of double emulsions [17,18,19]. In previous studies, the double emulsion delivery system based on calcium alginate hydrogel beads was developed and used to improve the stability of double emulsions [20]. However, upon application, it was observed that the hydrogel beads based on calcium alginate exhibited limited spreadability and suboptimal performance in application.

In the past decade, an emerging method for enhancing the stability of double emulsions has garnered increasing attention. This method involves synergistically combining two strategies: firstly, reinforcing the oil phase of double emulsions, and secondly, preventing coalescence between oil droplets [17,21,22]. The widely adopted approach involves initially reinforcing the oil phase of double emulsions with solid lipids, followed by stabilizing the double emulsions within hydrogel beads [17,23,24]. Chitosan, as a natural polysaccharide, inherently exhibits adhesive properties and enhances penetration, which makes it a widely utilized natural polymer in transdermal delivery systems [25]. Therefore, in this study, chitosan was selected as the gelling agent for fabricating hydrogel beads due to its unique properties and suitability for topical application. The ability of chitosan to form ionic gels with tripolyphosphate (TPP) is strongly influenced by its molecular weight [26]. As the molecular weight of chitosan increases, more chain entanglements occur, leading to an enhanced ability to form gels and a higher strength of the resulting gels.

The main purpose of this study is to develop a chitosan-based double emulsion-filled hydrogel bead system for topical delivery. Vitamin C, a natural antioxidant active ingredient widely used in personal care products, was used as a model hydrophilic active ingredient to prepare multiple emulsions and chitosan hydrogel beads for immobilizing multiple emulsions. Firstly, the leakage of active ingredients during the preparation of hydrogel beads is investigated. Secondly, the microscopic morphology of the system was studied, and the stability of this system was investigated. Further, the influence of chitosan hydrogel beads on the skin permeation behavior of double emulsions was investigated, with a focus on exploring the related mechanisms. On this basis, the performance in delivering hydrophobic active ingredients was also investigated.

## 2. Results and Discussion

### 2.1. Characterization of the Original (W/O/W) Double Emulsions

**Figure 1:**
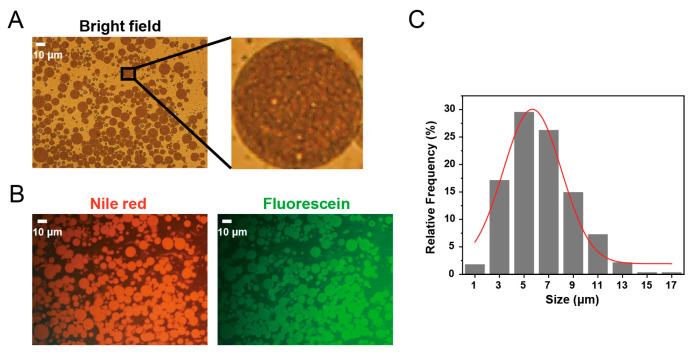
(**A**) Optical microscopic image of double emulsions. (**B**) Fluorescence microscopic images of double emulsions. (**C**) Size distribution of double emulsions (calculated using the image analysis method).

In the present study, acetylated monoglyceride (a type of semi-solid lipid) was added to the oil phase of the double emulsions to reinforce the stability of the oil phase and prevent aggregation of the inner aqueous phase droplets. The encapsulation efficiency of the initial double emulsions was (87.37 ± 4.16)%, as determined by the ultrafiltration method. The high encapsulation efficiency might be related to the relatively mild secondary emulsification process employed in the present study. The pH value of the initial double emulsions was 7.03 ± 0.18. As shown in Figure 1A, the bright field image of freshly prepared double emulsions revealed that the double emulsion droplets exhibited uniform spherical morphology and demonstrated excellent dispersion. The local amplification image showed numerous small and relatively uniform inner aqueous phase droplets within the larger oil droplets, without the presence of one or several large droplets within the oil droplets. To investigate the microscopic morphology of the original double emulsions, the middle oil phase and inner aqueous phase were labeled with the hydrophobic dye Nile Red and hydrophilic dye sodium fluorescein, respectively. As shown in Figure 1B, the fluorescence image confirmed that both the oil phase and the inner aqueous phase were uniformly distributed within the oil droplets. The microstructure suggested that the freshly prepared double emulsions exhibited the characteristics of a (W/O/W) double structure and aligned with the microscopic morphology feature of C-type double emulsions [27]. Compared with A-type double emulsions (containing only one large internal aqueous droplet) and B-type double emulsions (containing several internal aqueous droplets), C-type double emulsions are considered more effective in stabilizing double emulsions and ensuring long-term encapsulation of hydrophilic active ingredients [28]. The size distribution of double emulsions is shown in Figure 1C. The mean particle size of the emulsion was determined to be 6.38 ± 2.67 μm, the median particle size (D50) was 6.608 μm, and the Span value was 0.981.

### 2.2. Incorporation of Double Emulsions in Chitosan Hydrogel Beads

**Figure 2:**
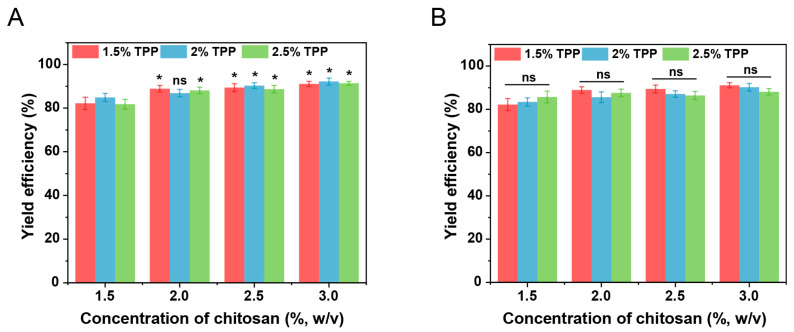
(**A**) The effect of chitosan concentration and sodium tripolyphosphate (TPP) concentration on the yield efficiency (crosslinking time fixed at 20 min); * statistically significant differences from 1.5% chitosan concentration group at the same TPP concentration (*p* < 0.05); ns indicates no significance (n = 3). (**B**) The effect of chitosan concentration and crosslinking time on the yield efficiency (sodium tripolyphosphate concentration fixed at 1.5%, *w*/*v*); ns indicates no significance at the same chitosan concentration (n = 3).

The leakage of the internal aqueous phase leads to the leakage of hydrophilic active ingredients and the disappearance of the multiple structures, which is the main type of destabilization of double emulsions. To assess the feasibility of incorporating double emulsions within chitosan hydrogel beads, the encapsulation of double emulsions-filled chitosan hydrogel beads for model hydrophilic active ingredients (vitamin C) in double emulsions was investigated through yield efficiency. The chitosan concentration, crosslinking agent (sodium tripolyphosphate, TPP) concentration, and crosslinking time might influence the leakage of active ingredients during the crosslinking process. Preliminary experiments found that an insufficient chitosan concentration was incapable of forming structurally intact spherical gels. Therefore, the concentrations that can form hydrogel beads were investigated through yield efficiency. As shown in Figure 2A, compared with higher concentrations, the yield efficiency was relatively low when the chitosan concentration was 1.5% (*w*/*v*). At a lower chitosan concentration, after the mixture solution was dropped into the crosslinking solution, in addition to forming hydrogel beads, a small number of small fragments were observed upon contact with the crosslinking agent. These fragments subsequently entered the crosslinking and washing solutions during the cleaning and recovery process, leading to reduced yield efficiency. When the chitosan concentration exceeded 2% (*w*/*v*), no significant changes in yield efficiency were observed as the chitosan concentration increased. This might be due to the relatively low surface activity of chitosan, which had no effect on the stability of the oil–water interface of double emulsions during preparation. Under the same chitosan concentration, the crosslinking agent concentration had no significant effect on the yield efficiency. As shown in Figure 2B, under different crosslinking time conditions, there were no significant differences in the yield efficiency. This may be attributed to the fact that the leaked hydrophilic active ingredients primarily originated from the free hydrophilic active ingredients in the outer water phase, which were rapidly released into the crosslinking solution during the crosslinking process. This result further confirms that the crosslinking process did not cause the leakage of the internal aqueous phase of the double emulsions, thereby validating the feasibility of the preparation technology.

### 2.3. Characterization of Double Emulsions-Filled Chitosan Hydrogel Beads

**Figure 3:**
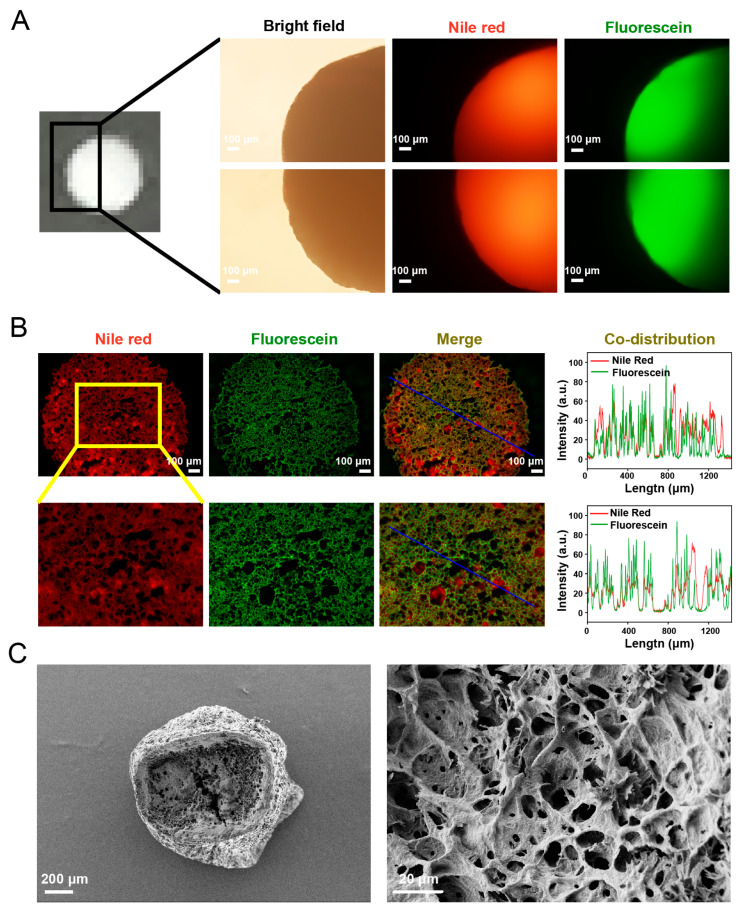
(**A**) The overall morphology of double emulsions-filled chitosan hydrogel beads loaded with vitamin C and fluorescence microscopic images of double emulsions-filled chitosan hydrogel beads (two different positions of the same beads). (**B**) Fluorescence microscopic images of double emulsions-filled chitosan hydrogel beads (frozen section). Fluorescence co-distribution characteristics were quantified based on the blue lines in merged images. (**C**) Overall (**left**) and sectional (**right**) SEM images of double emulsions-filled chitosan hydrogel beads.

The overall morphology of two different positions of the same double emulsions-filled chitosan hydrogel beads under a light microscope is shown in Figure 3A. The bright-field image revealed that hydrogel beads exhibited a spherical shape with a smooth surface. The diameter of the double emulsions-filled chitosan hydrogel beads was measured to be 2.31 ± 0.14 mm. Nile Red and sodium fluorescein were used to label the oil phase and the inner aqueous phase of the double emulsions within hydrogel beads, respectively. The fluorescence images demonstrated that Nile Red was distributed throughout the hydrogel beads, suggesting that double emulsions were uniformly distributed throughout the beads. The observed non-uniform distribution of fluorescein might be caused by the excessive thickness of the sample. By comparing the fluorescence images of different positions of the same bead, it could be found that fluorescein was also distributed throughout the beads, indicating a uniform distribution of the inner aqueous phase within the beads. Figure 3B shows the fluorescence microscopic images of the frozen section of the beads and the co-distribution analysis results of Nile Red and fluorescein. The distribution of Nile Red and fluorescein in the section indicated that the double emulsion droplets were distributed throughout the beads. Moreover, Nile Red and fluorescein exhibited a network-like distribution within the beads, which might be related to the three-dimensional network structure of the chitosan gel matrix. The distribution of the double emulsions appeared to be constrained by this network structure. The merged images and the co-distribution analysis revealed that fluorescein and Nile Red had obvious overlapping and co-distribution characteristics, suggesting that fluorescein in the inner aqueous phase was still encapsulated in the double emulsion droplets and distributed within the hydrogel matrix along with the oil phase. The above results revealed that after incorporating the double emulsions into hydrogel beads, the inner aqueous phase was still encapsulated in the oil droplets, indicating the stability of double emulsions within beads. This was consistent with the result of the yield efficiency, confirming the feasibility of the preparation technology. The SEM images show that the interior of the hydrogel beads consists of a loose and porous network-like structure (Figure 3C). The void fraction of double emulsions-filled chitosan hydrogel beads was measured to be (94.29 ± 0.08)%. This result also indicated that the hydrogel beads possessed a porous structural feature.

### 2.4. Stability Study

**Figure 4:**
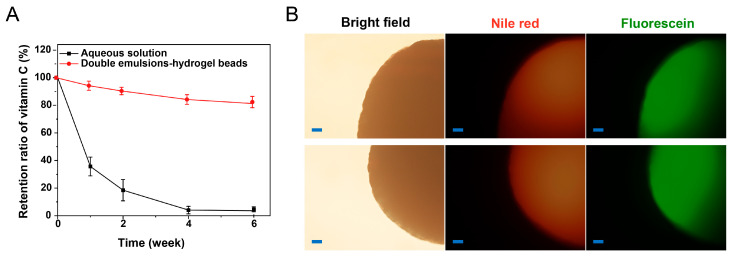
(**A**) Vitamin C retention ratio of vitamin C aqueous solution and double emulsions-filled chitosan hydrogel beads loaded with vitamin C during the storage 20 °C. (**B**) The appearance of double emulsions-filled chitosan hydrogel beads after 6 weeks of storage at 20 °C. Scale bars: 100 μm.

The stability of vitamin C is an important factor affecting its application. The retention rate of vitamin C in aqueous solution and double emulsions-filled chitosan hydrogel beads during the storage process is shown in Figure 4A. The results showed that compared with the vitamin C aqueous solution, vitamin C in the double emulsions-filled chitosan hydrogel beads had better stability. This indicated that the delivery system had the effect of improving the stability of the hydrophilic active ingredients during the storage process. After 6 weeks of storage, the distribution of Nile Red and fluorescein in the hydrogel beads also indicated that this system has good stability and can avoid the leakage of the internal aqueous phase during the storage process (Figure 4B). In this study, solid lipids were added to the oil phase of the double emulsions to strengthen the multiple structures. At the same time, the double emulsions were fixed in the hydrogel beads to synergistically stabilize the multiple structures. The emulsions with a stable multiple structure can effectively isolate the influence of the external environment on the hydrophilic active ingredients in the internal aqueous phase, thereby improving the stability of the hydrophilic active ingredients. Previous studies have also reported that double emulsions with a stable structure could improve the storage stability of vitamin C [9,20]. The morphology of double emulsion-filled chitosan hydrogel beads remained unchanged, and the beads maintained a white color throughout the 6-week storage period (Table 1). Additionally, there were no significant changes in diameter (*p* > 0.05), indicating that the hydrogel beads remained intact without degradation over a 6-week storage period.

### 2.5. Skin Permeation of Hydrophilic Active Ingredients

**Figure 5:**
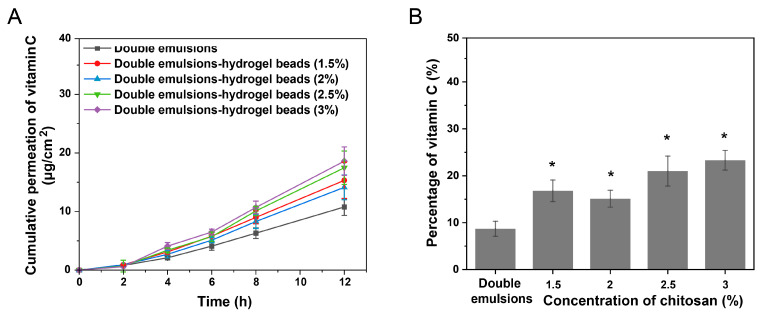
(**A**) The cumulative amount of vitamin C penetrating through the skin from double emulsions and double emulsions-filled chitosan hydrogel beads prepared with different chitosan concentrations. (**B**) The amount of vitamin C accumulating in the skin from double emulsions and double emulsions-filled chitosan hydrogel beads prepared with different chitosan concentrations after 12 h of treatment. * Statistically significant differences from double emulsions (*p* < 0.05) (n = 6).

In the present study, the skin permeation ability of the initial double emulsions and double emulsions-filled chitosan hydrogel beads prepared using different chitosan concentrations was investigated through in vitro skin penetration experiments. Chitosan is a highly biocompatible cationic polysaccharide that usually does not cause inflammatory reactions. Chitosan also has anti-inflammatory effects and is currently widely used in the preparation of skin and wound anti-inflammatory materials [29]. During skin permeation experiments, the hydrogel beads were topically applied to form a thin film on the skin surface. After completion of permeation studies, residual formulations were removed by rinsing with physiological saline. The cumulative permeation amounts of vitamin C in different systems after 12 h of treatment are shown in Figure 5A. The amounts of vitamin C accumulating in the skin after 12 h of treatment are shown in Figure 5B. Permeation parameters (flux J, lag time, permeability coefficient) were also calculated and shown in Table 2.

The results indicated that the vitamin C loaded in the initial double emulsions could be released from the inner aqueous phase and then penetrate into the skin. After the double emulsions were applied to the skin surface, due to changes in the external environment, the active ingredients in the inner aqueous phase were released into the outer aqueous phase and then penetrated into the skin. Due to the barrier effect of the stratum corneum on hydrophilic active ingredients, the process of the released hydrophilic active ingredients penetrating into the stratum corneum might be the rate-limiting step for the skin permeation of hydrophilic active ingredients in double emulsions [30,31]. Under the four chitosan concentrations, the cumulative permeation amount of vitamin C in the hydrogel beads was higher than that in the double emulsion group. When the chitosan concentration was 1.5% and 2% (*w*/*v*), the difference was not significant (*p* > 0.05), while when the chitosan concentration was 2.5% and 3% (*w*/*v*), there was a significant difference (*p* < 0.05). When the concentration of chitosan was 3% (*w*/*v*), the permeation coefficient (*K_p_*) of vitamin C from the hydrogel beads exhibited an increase (1.7-fold) compared with the double emulsions. Under the four chitosan concentrations, the amount of vitamin C accumulated in the skin from double emulsion-filled chitosan hydrogel beads was significantly higher than that observed in the double emulsions group. The above results indicated that using hydrogel beads to stabilize double emulsions could promote the skin permeation and retention of the active ingredients. This may be because the hydrogels could increase the water content of the stratum corneum [32]. The stratum corneum with a higher water content is conducive to the permeation of hydrophilic active ingredients [33]. In addition, it has been reported that polymers and hydrogels could improve the adhesion performance of the delivery system, thereby promoting the skin permeation ability of active ingredients [34,35].

### 2.6. Skin Permeation Behavior Study Based on ATR-FTIR

To study the skin permeation behavior and mechanism, the effect of the delivery systems on the skin was investigated using ATR-FTIR. ATR-FTIR is a commonly used method for analyzing the structure of the stratum corneum and could reveal the influence of transdermal delivery systems on the lipid structure in the stratum corneum at the molecular level. The orderly arranged lipids in the stratum corneum are usually regarded as the main barrier for the skin permeation of active ingredients. The most important information on lipids in the spectrum is the absorption peaks near 2920 cm^−1^ (asymmetric stretching vibration of CH_2_) and 2850 cm^−1^ (symmetric stretching vibration of CH_2_), which can reflect the conformation of lipids in the stratum corneum.

**Figure 6:**
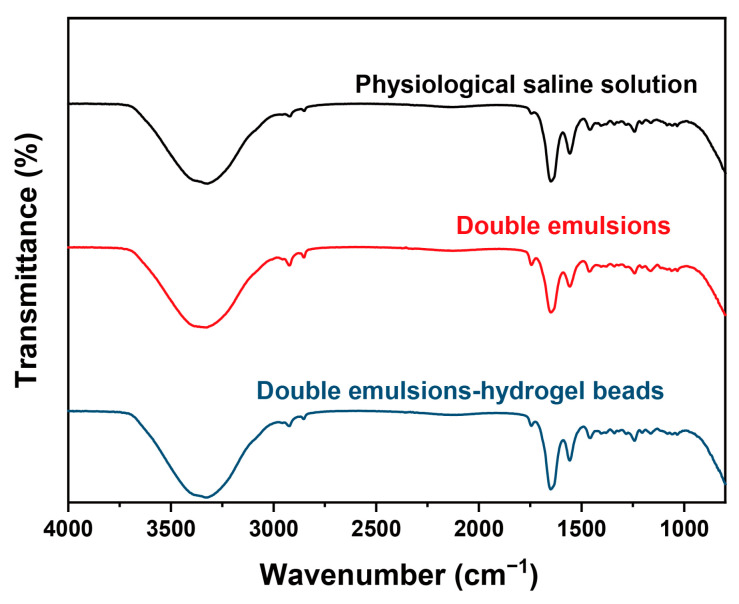
The ATR-FTIR spectra of the skins after treatment with physiological saline solution, double emulsions, or double emulsions-filled chitosan hydrogel beads (chitosan concentration 3%, *w*/*v*) for 12 h.

In this study, the skin treated with physiological saline solution was selected as the control group. The results are shown in Figure 6 and Table 3. In the physiological saline solution group, the asymmetric stretching vibration peak of CH_2_ and the symmetric stretching vibration peak of CH_2_ were located at 2918.86 cm^−1^ and 2850.46 cm^−1^, respectively. While in the double emulsion group, these two peaks shifted to 2922.68 cm^−1^ and 2852.38 cm^−1^, respectively. This result suggested that double emulsions could disrupt the conformation of lipids in the stratum corneum and increase the fluidity of lipids [36]. This might be attributed to the fact that the exogenous lipids in the oil phase of double emulsions can coexist with the endogenous lipids in the stratum corneum as penetration enhancers and disrupt the orderly arrangement of endogenous lipids. Similar to the double emulsion group, the characteristic peaks of lipids in the double emulsion-filled chitosan hydrogel beads group also shifted significantly, indicating that double emulsion-filled chitosan hydrogel beads also have the effect of influencing the lipid structure in the stratum corneum.

### 2.7. Skin Permeation Behavior Study Based on Fluorescent Probes

To further study the effect of hydrogel beads on the transdermal behavior of double emulsions, the oil phase of double emulsions was labeled with the hydrophobic fluorescent dye Nile Red, while the hydrophilic fluorescent dye sodium fluorescein was loaded in the inner aqueous phase of double emulsions. The method of using both hydrophobic and hydrophilic dyes to label different parts of the transdermal delivery system can visually clarify the transdermal behavior of the complex delivery system [37].

**Figure 7:**
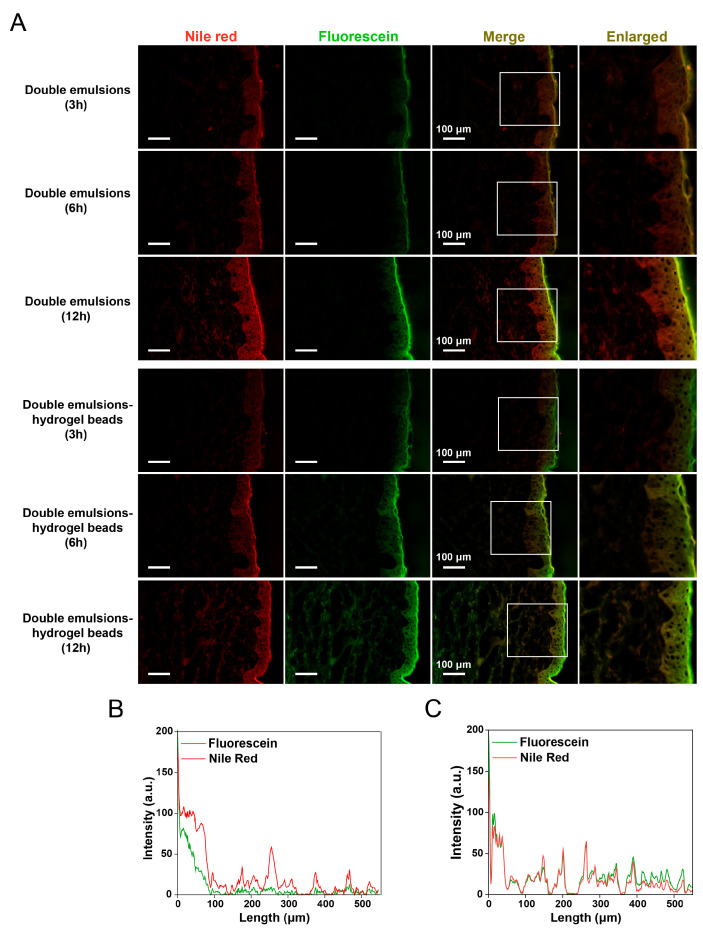
(**A**) Fluorescence microscopic images of skins after treatment with double emulsions and double emulsions-filled chitosan hydrogel beads (chitosan concentration 3%, *w*/*v*) for 3 h, 6 h, and 12 h. (**B**) Fluorescence intensity profiles across skin depth (double emulsions, 12 h). (**C**) Fluorescence intensity profiles across skin depth (double emulsions-filled chitosan hydrogel beads, 12 h).

As shown in Figure 7A, both the double emulsions and double emulsion-filled chitosan hydrogel beads facilitated the release of sodium fluorescein from the inner aqueous phase into the skin, while Nile Red from the oil phase also successfully penetrated into the skin. Due to the large size of double emulsions, they cannot directly penetrate into the stratum corneum. The permeation of the dye in the oil phase might occur along with the permeation of other components of the double emulsions into the skin. First, the hydrophilic emulsifiers on the surface of the double emulsions might penetrate into the skin and have a certain permeation-promoting effect [38,39]. In addition, the unsaturated fatty acids in corn oil may penetrate the skin and alter the lipid conformation on the surface of the stratum corneum, thereby enhancing the permeation of hydrophobic components from the oil phase [40,41,42].

With the prolongation of treatment time, fluorescent dyes were observed to penetrate deeper into the skin. Notably, significant differences were observed between the double emulsion group and the double emulsion-loaded chitosan hydrogel beads group. After treating the skin with double emulsions, the permeation depth of Nile Red was significantly higher than that of sodium fluorescein, and the accumulation of sodium fluorescein in the deep skin was less (Figure 7B). After treating the skin with double emulsion-filled chitosan hydrogel beads, sodium fluorescein could also penetrate into the deep skin, and there was an obvious accumulation of sodium fluorescein in the epidermis and dermis after 12 h (Figure 7C). These results indicated that chitosan hydrogel could modify the transdermal behavior of double emulsions and demonstrate a more significant enhancement in the permeation of hydrophilic active ingredients from the inner aqueous phase. The obstruction of transdermal permeation mainly arises from the dense stratum corneum. Due to the highly hydrophobic nature of the stratum corneum, hydrophilic active ingredients face significantly greater challenges in permeation than hydrophobic active ingredients. Additionally, there are significant differences in the permeation mechanisms between hydrophobic and hydrophilic components within the stratum corneum. The transdermal permeation of hydrophilic active ingredients mainly occurs through the hydrophilic pores in the stratum corneum, and this route depends on the water content of the skin. Therefore, a highly hydrated environment could promote the transdermal permeation of hydrophilic components [33,43]. Hydrogels are capable of increasing the water content of the stratum corneum, which may consequently enhance their role in facilitating the skin permeation of hydrophilic active ingredients. In the early stages of application, the hydrogel system enhanced skin water content. However, as the contact time between the hydrogel and the skin increases, the hydrogel might pull moisture from the skin, leading to a reduction in hydration levels. In this study, the hydrogel system contained the oil phase of double emulsions, which formed an occlusive oil film on the skin surface to inhibit transepidermal water loss. Additionally, the incorporation of solid lipids in the oil phase further enhanced the occlusive effect of the system, thereby reducing water evaporation. These mechanisms collectively extended the duration of the hydrogel system’s beneficial effects. During the application process, double emulsions might gradually transform into a simple water-in-oil emulsion structure on the skin surface due to factors such as the evaporation of the external aqueous phase and phase transition [44]. The initial direct contact mode of the stratum corneum with the continuous aqueous phase changed to the contact mode with the continuous oil phase, which was not conducive to increasing the water content of the stratum corneum. However, the hydrophilic polymer network structure in the external aqueous phase of gelled double emulsions can effectively suppress structural alterations induced by the loss of the external aqueous phase. This prolonged the contact duration between the stratum corneum and the aqueous phase, thereby enhancing the water content of the stratum corneum to a certain extent and facilitating the penetration of hydrophilic active ingredients [39].

### 2.8. Skin Permeation of Hydrophobic Active Ingredients

In the above studies, the influence and mechanism of chitosan hydrogel beads on the skin penetration of hydrophilic active ingredients in double emulsions have been elucidated. Considering that double emulsions are also an important co-delivery system for both hydrophilic and hydrophobic active ingredients, the influence on the permeation of hydrophobic active ingredients was also investigated. In this study, quercetin was selected as the model hydrophobic active ingredient and was added to the oil phase of the double emulsions during the preparation process. The oil phase of the double emulsion consisted of a blend of acetylated monoglycerides and corn oil. The acetyl groups in acetylated monoglycerides acted as strong hydrogen–bond acceptors, forming stable hydrogen–bond networks with phenolic hydroxyl groups of quercetin, thereby enhancing the solubility of quercetin in the oil phase. Furthermore, the mixed lipid system synergistically improved quercetin solubility. Acetylated monoglycerides provided hydrogen–bond acceptors to solubilize the polar phenolic hydroxyl groups, and corn oil offered long alkyl chains to accommodate the hydrophobic benzene rings. This combination optimized the molecular microenvironment of the oil phase, significantly enhancing quercetin solubility. Through solubility experiments, we determined quercetin’s solubility in this oil phase to be about 8.5 mg/g, substantially higher than in corn oil alone. This enhanced solubility confirmed the capability of this system for effective quercetin loading.

**Figure 8:**
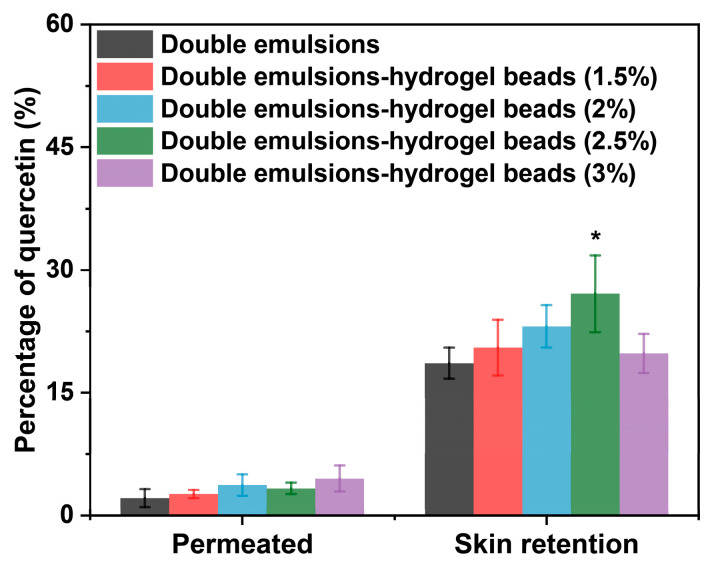
Amount of hydrophobic active ingredients (quercetin) penetrating through the skin and accumulating in the skin after 12 h treatment with quercetin-loaded double emulsions and double emulsions-filled chitosan hydrogel beads prepared with different chitosan concentrations. * Statistically significant differences from double emulsions (*p* < 0.05). (n = 6).

As shown in Figure 8, chitosan hydrogel beads did not hinder the skin penetration of the hydrophobic active ingredient in the oil phase of the multiple emulsions, which was consistent with the research results based on fluorescent dyes. Under the specific chitosan concentration condition (2.5%, *w*/*v*), chitosan hydrogel beads exhibited a promoting effect on the permeation of the hydrophobic active ingredient in the double emulsions. This might be related to the fact that chitosan hydrogel could affect the barrier function of the stratum corneum and thereby promote the penetration of hydrophobic active ingredients. These results suggested the potential application of double emulsions-filled chitosan hydrogel beads in the co-delivery of hydrophilic and hydrophobic active ingredients.

## 3. Conclusions

In this study, double emulsions loaded with a model hydrophilic active ingredient (vitamin C) were prepared using a two-step emulsification method. The encapsulation efficiency of the initial double emulsions was (87.37 ± 4.16)%. Subsequently, double emulsions-filled chitosan hydrogel beads were fabricated via an extrusion technique. The results revealed that both the oil phase and the inner aqueous phase of the double emulsions were uniformly distributed throughout the hydrogel beads. The yield efficiency was above 80%, indicating that the incorporated emulsions maintained effective encapsulation of the inner aqueous phase. Stability studies demonstrated that, after incorporating the hydrophilic active ingredient (vitamin C) into this system, the retention ratio after six weeks of storage was observed to increase by 77.6%. Furthermore, this system disrupted the orderliness of lipid structures in the stratum corneum, thereby enhancing the permeation of hydrophilic active ingredients. When the concentration of chitosan was 3% (*w*/*v*), the permeation coefficient of vitamin C from hydrogel beads was observed to increase by 1.7-fold compared with that from double emulsions. Meanwhile, double emulsions-filled chitosan hydrogel beads also obtained a 2.6-fold increase in skin uptake of vitamin C. The permeation behavior studies based on fluorescent dyes indicated that chitosan hydrogel beads altered the permeation behavior of double emulsions. Compared to the influence on the oil phase, chitosan hydrogel beads more effectively promoted the permeation of hydrophilic active ingredients from the inner aqueous phase. Finally, the impact of chitosan hydrogel beads on the skin permeation of hydrophobic active ingredients loaded in double emulsions was also investigated. The results confirmed that this system was also applicable to the delivery of hydrophobic active ingredients. Overall, double emulsions-filled chitosan hydrogel beads demonstrate potential as an effective active ingredient delivery system with broad application prospects.

## 4. Materials and Methods

### 4.1. Materials

Corn oil was purchased from Jinhai Food Industry Co., Ltd. (Qinghuangdao, China). Acetylated monoglyceride was purchased from Zhengtong Food Technology Co., Ltd. (Zhengzhou, China). PEG-30 Dipolyhydroxystearate (P135) was obtained from Croda (East Yorkshire, UK). Tween 80 was purchased from Guangzhou Runhua Chemical Co., Ltd. (Guangzhou, China). Ascorbic acid and sodium tripolyphosphate (TPP) were purchased from Shanghai Titan Technology Co., Ltd. (Shanghai, China). Chitosan hydrochloride (degree of deacetylation: 91.5%; molecular weight: about 200,000) was purchased from Gebeisi Food Additive Co., Ltd. (Zhengzhou, China). Nile Red was purchased from Sigma-Aldrich (St. Louis, MO, USA). Sodium fluorescein was purchased from TCI Development Co., Ltd. (Shanghai, China).

### 4.2. Preparation of Double Emulsions

Purified water was used as the inner aqueous phase. For the preparation of vitamin C-loaded double emulsions, a certain amount of vitamin C (100 mg/mL) was weighed and dissolved in purified water to obtain the inner aqueous phase. For the preparation of fluorescein-labeled double emulsions, the hydrophobic fluorescent dye sodium fluorescein was added to the inner aqueous phase. The lipophilic emulsifier (P135, 6.25%, *w*/*w*), solid lipid (ACETEM, 18.75%, *w*/*w*), and liquid lipid (corn oil, 75%, *w*/*w*) were stirred and mixed completely at 60 °C to form the lipid phase. For the preparation of Nile Red-labeled double emulsions, the hydrophobic fluorescent dye Nile Red was added to the lipid phase. A certain amount of hydrophilic emulsifier (Tween 80, 5%, *w*/*w*) was weighed and fully dissolved in purified water to form the outer aqueous phase.

The double emulsions were prepared using a modified two-step emulsification method described in our previous study [45]. The first emulsification process is as follows: the inner aqueous phase (20%, *w*/*w*) was gently added to the lipid phase (80%, *w*/*w*) at 60 °C under continuous stirring at 800 rpm using an overhead stirrer (RW20, IKA, Staufen, Germany). Subsequently, the mixture was further stirred at 60 °C for 5 min and homogenized for 2 min at 10,000 rpm using a preheated high-shear emulsifier (FA25, FLUKO Technology, Essen, Germany) to obtain the (W/O) primary emulsion. The (W/O) primary emulsion was transferred to a water bath at 35 °C and cooled to 35 °C under the condition of continuous stirring at 800 rpm.

The second emulsification process is as follows: Firstly, the outer aqueous phase was heated to 35 °C. Subsequently, the pre-cooled (W/O) primary emulsion (30%, *w*/*w*) at 35 °C was gently introduced into the outer aqueous phase (70%, *w*/*w*) under stirring at 400 rpm and further dispersed for 3 min at 35 °C. Finally, the (W/O/W) double emulsions were obtained after cooling at room temperature.

### 4.3. Preparation of Double Emulsions-Filled Chitosan Hydrogel Beads

The double emulsions-filled chitosan hydrogel beads were prepared by the extrusion method combined with the ionic gelation method [17]. A precise amount of chitosan hydrochloride was accurately weighed and dissolved in purified water under continuous stirring until complete dissolution. Subsequently, ultrasonic treatment was applied to eliminate any bubbles in the chitosan solution, yielding chitosan stock solutions at various concentrations. The above (W/O/W) double emulsions were mixed with chitosan solutions (3%, 4%, 5%, 6% *w*/*v*) at a 1:1 volume ratio and stirred at 200 rpm for 5 min to prepare double emulsion–chitosan sols with varying chitosan concentrations (1.5%, 2%, 2.5%, 3% *w*/*v*). Then the sols were dropped dropwise into the crosslinked fluid (sodium tripolyphosphate solution) using a programmable syringe pump (LSP01-1A, Longer, Baoding, China). The flow rate of the injection pump was set at 0.2 mL/min, and the distance from the needle to the sodium tripolyphosphate solution was 10 cm. The formed chitosan hydrogel beads were placed in the crosslinked fluid and continued to be crosslinked at room temperature for a period of time. After the crosslinking process was completed, the crosslinked fluid was collected. Subsequently, the chitosan hydrogel beads were washed twice with purified water, and the washing solution was also carefully collected. Finally, the collected crosslinked fluid and washing water were mixed for encapsulation efficiency determination.

When investigating the yield efficiency, the impacts of varying chitosan concentrations (1.5%, 2%, 2.5%, 3%, *w*/*v*), sodium tripolyphosphate concentrations (1.5%, 2%, 2.5%, *w*/*v*), and continuous crosslinking times (20, 30, 40 min) on the encapsulation efficiency were examined. In the other sections, the concentration of sodium tripolyphosphate was fixed at 1.5% (*w*/*v*), and the crosslinking time was set to 20 min. When the chitosan concentration was 1% and 0.5% (*w*/*v*), droplets failed to solidify upon contacting the TPP crosslinking solution, resulting in diffuse dispersion without bead formation. When the chitosan concentration was 3.5% and 4% (*w*/*v*), irregular bead morphology with pronounced tail formation occurred.

### 4.4. Encapsulation Efficiency of Double Emulsions

The ultrafiltration centrifugation method was employed to separate the outer aqueous phase and quantify encapsulation efficiency. The amount of vitamin C transferred from the inner aqueous phase to the outer aqueous phase during secondary emulsification was considered free vitamin C. An amount of 400 μL of freshly prepared double emulsions was added to an ultrafiltration centrifugation tube (molecular weight cut-off: 30 kDa, Millipore, Billerica, MA, USA) and centrifuged at 10,000 rpm for 30 min. All the ultrafiltrate from the lower layer of the ultrafiltration centrifugation tube was collected, and the content of vitamin C in the ultrafiltrate was quantified by ultraviolet spectrophotometry at 256 nm. Finally, the encapsulation efficiency (EE_DE_) of the double emulsions was calculated according to the following formula:

(1) $${\mathrm{EE}}_{\mathrm{DE}}\;(\%)=(1-\frac{W_{F}\;}{W_{T}})\;\times \;100\%$$

where W_F_ is the content of vitamin C in the ultrafiltrate (free vitamin C), and W_T_ is the total content of vitamin C in the double emulsion system.

The method was verified through the recovery experiment. Vitamin C solution with different concentrations (1 mg/mL, 10 mg/mL, 100 mg/mL) was added into the ultrafiltration centrifugation tube and centrifuged at 10,000 rpm for 30 min. Recovery averaged (96.7 ± 1.5)% (n = 9 across 3 concentrations), confirming minimal retention.

### 4.5. Yield Efficiency of Double Emulsions-Filled Chitosan Hydrogel Beads

The yield efficiency (YE) of double emulsions-filled chitosan hydrogel beads was calculated by determining the amount of the model active ingredient (vitamin C) leaked from the hydrogel beads during the crosslinking process. After the preparation of the chitosan hydrogel beads, the content of vitamin C in the mixed solution of the crosslinking solution and washing water (representing the leakage amount of vitamin C) was measured. Subsequently, the yield efficiency (YE) of the systems was calculated using the following formula:

(2) $$\mathrm{YE}\;(\%)=(1\;-\;\frac{W_{M}\;}{W_{T}})\;\times \;100\%$$

where W_M_ is the amount of vitamin C in the mixed solution of the crosslinking solution and washing water, and W_T_ represents the total amount of vitamin C.

### 4.6. Analysis of Diameter and Void Fraction

Following the preparation of the beads, a total of 60 beads were assessed to determine the average diameter. The void fraction of the beads was analyzed using the method described in prior studies [46]. The prepared beads underwent dehydration through freeze-drying, and the weight of the dried beads was subsequently recorded. Thereafter, the void fraction of the beads was calculated utilizing the following formula:

(3) $$\mathrm{void}\;\mathrm{fraction}\;(\%)=\frac{V-(M/\rho )}{V}\;\times \;100\%$$

where V is the volume of beads, M is the weight of dried beads, and ρ is the density of the mixture of double emulsions and chitosan.

### 4.7. Analysis of Morphology

The microstructure of double emulsions was observed through a fluorescence microscope (IX73, Olympus, Tokyo, Japan). For the double emulsions used in morphology observation, sodium fluorescein was incorporated into the inner aqueous phase during preparation, while Nile Red was added to the oil phase. Following sample preparation, the morphology of the double emulsions was observed under both bright-field and fluorescence microscopy.

The double emulsions-filled chitosan hydrogel beads labeled with the fluorescent dyes were prepared for microscopic morphology observation. A single fluorescent-labeled bead was placed on a glass slide, and its edge was observed under both bright-field and fluorescence microscopy. Additionally, the bead was subjected to cryo-sectioning, and the resulting section was subsequently observed under fluorescence microscopy.

Overall and sectional morphology of double emulsions-filled chitosan hydrogel beads was also observed using a scanning electron microscope (SEM) (Ul-tra Plus, Zeiss, Oberkochen, Germany), according to previous studies [47]. The bead was washed with hexane to remove all oil excipients. Then, beads were fixed onto a sample stage using double-sided carbon adhesive tape and then observed using SEM.

### 4.8. Storage Stability

The aqueous solution of vitamin C (3 mg/mL) and the quantitatively divided double emulsions-filled chitosan hydrogel beads loaded with vitamin C were sealed in glass vials and stored at 20 °C. The vitamin C content in the system was measured at 0, 1, 2, 4, and 6 weeks, and the retention rate of vitamin C was subsequently calculated.

### 4.9. Skin Permeation Study

The in vitro skin permeation study was performed using the vertical Franz diffusion method [9]. The porcine skin was selected as the model skin in this study and obtained from Linxi Jingde Co., Ltd. (Xingtai, China). Skin samples were obtained from a total of six distinct pigs. The animal study protocol was approved by the Laboratory Animal Center of Nantong University. For each experiment, there were six repetitions. The skin removal process was performed manually, and hair was removed with a razor. The intact pig skins had a thickness ranging from 0.8 to 1.0 mm. After isolation, the pig skins were cut into pieces slightly larger than the diffusion cell and washed with saline solution. Prior to experimentation, the initial double emulsions were mixed with an equal volume of purified water to ensure comparability across different samples. The receiving chambers were filled with receiving medium (saline solution), and the prepared, isolated porcine skins were secured between the receiving chamber and the donor chamber, with the stratum corneum facing the donor chamber. During the operation, the creation of bubbles between the receiving medium and the skin was avoided. The volume of the receiving chamber is 6.5 mL, and the effective diffusion area of the skin is 2.8 cm^2^. Subsequently, the diluted double emulsions and a specific quantity of double emulsions-filled chitosan hydrogel beads (equivalent to 300 μL of the initial double emulsions) were introduced into the donor chambers. The samples were gently spread and pressed to ensure even distribution on the skin surface and full contact with the skin. Throughout the permeation experiment, the temperature was maintained at 32 °C, and the magnetic stirring speed in the receiving chamber was set to 100 rpm. At the selected time point, an appropriate amount of the medium was taken out from the receiving chamber and analyzed by the HPLC method for the determination of the permeation amount, and an equal amount of the medium was replenished. After the skin permeation was completed, the skin was removed from the diffusion cells and rinsed with physiological saline to remove the residual samples. The skins were then cut into pieces and immersed in PBS solution for ultrasonic treatment for 30 min and then centrifuged at 10,000 rpm for 10 min. The supernatant was collected and analyzed by the HPLC method for the determination of the skin accumulation amount.

### 4.10. Skin Permeation Study Based on ATR-FTIR

ATR-FTIR (Attenuated Total Reflection Fourier Transform Infrared Spectroscopy) was employed to investigate the effects of double emulsions and chitosan hydrogel beads on the lipid structure of the stratum corneum. The normal saline was used as the control. The skin permeation was conducted following the procedures outlined in Section 4.8. After completion of the skin permeation study, the skin samples were excised, and residual samples on the skin surface were carefully removed by rinsing with normal saline. Both sides of the skin were subsequently dried using filter paper. The dried skin samples were then placed on the ZnSe crystal of the ATR accessory for analysis. The scanning parameters were set as follows: a wavenumber range of 4000–800 cm^−1^ and 32 scans.

### 4.11. Skin Permeation Study Based on Fluorescent Probes

During the preparation of multiple emulsions, sodium fluorescein (5%, *w*/*w*) was incorporated into the inner aqueous phase, while Nile Red (0.2%, *w*/*w*) was added to the oil phase simultaneously. Subsequently, double emulsions-filled chitosan hydrogel beads were fabricated using this emulsion system. The skin permeation experiment was performed according to the procedure outlined in Section 4.8. Upon completion of the experiment, the skin samples were carefully excised, and any residual samples on the skin surface were thoroughly removed with physiological saline. The skin tissues were then cryo-embedded using OCT and sectioned at a thickness of 10 μm. Finally, the distribution of fluorescent dyes within the skin sections was observed under fluorescence microscopy.

### 4.12. Statistical Analysis

The results were expressed as means ± standard deviation. Normality was verified using Shapiro–Wilk tests. Data were analyzed using one-way ANOVA followed by Tukey’s post hoc test. Statistical significance was set at *p* < 0.05.

## Figures and Tables

**Table 1:** Stability studies of double emulsions-filled chitosan hydrogel beads loaded with vitamin C during storage at 20 °C.

Period	Morphology	Color	Diameter (mm)	Retention Ratio of Vitamin C (%)
0 week	Spherical and smooth	White	2.31 ± 0.14	100 ± 0.5
1 week	Spherical and smooth	White	2.34 ± 0.16	94.2 ± 3.3
2 week	Spherical and smooth	White	2.41 ± 0.21	90.4 ± 2.7
4 week	Spherical and smooth	White	2.39 ± 0.17	84.2 ± 3.5
6 week	Spherical and smooth	White	2.36 ± 0.19	81.3 ± 4.1

**Table 2:** Permeability parameters of vitamin C penetrating through the skin from double emulsions and double emulsions-filled chitosan hydrogel beads prepared with different chitosan concentrations.

		Flux (*J_ss_*) (μg/(cm^2^·h))	Lag Time (h)	Permeability Coefficient (Kp) (10^−4^ cm/h)
Double emulsions		1.089 ± 0.1370	2.165 ± 0.095	3.630 ± 0.456
Double emulsions- hydrogel beads	1.5%	1.538 ± 0.3585	2.027 ± 0.496	5.128 ± 1.196
	2%	1.445 ± 0.2125	2.271 ± 0.221	4.818 ± 0.708
	2.5%	1.801 ± 0.2970 *	2.394 ± 0.181	6.004 ± 0.990 *
	3%	1.856 ± 0.2430 *	2.117 ± 0.085	6.186 ± 0.809 *

* Statistically significant differences from the double emulsions group (*p* < 0.05). (n = 6).

**Table 3:** Characteristic ATR-FTIR absorption peaks of lipids in the skins after treatment with physiological saline solution, double emulsions, or double emulsions-filled chitosan hydrogel beads (chitosan concentration 3%, *w*/*v*) for 12 h.

	Asymmetric CH_2_ (cm^−1^)	Symmetric CH_2_ (cm^−1^)
Physiological saline solution	2918.86 ± 0.64	2850.46 ± 0.25
Double emulsions	2922.68 ± 0.52 *	2852.38 ± 0.71 *
Double emulsions-hydrogel beads	2923.95 ± 0.47 *	2852.87 ± 0.21 *

* Statistically significant differences from physiological saline solution (*p* < 0.05). (n = 6).

## Data Availability

The original contributions presented in this study are included in the article. Further inquiries can be directed to the corresponding authors.
